# Differences of Transport Activity of Arginine and Regulation on Neuronal Nitric Oxide Synthase and Oxidative Stress in Amyotrophic Lateral Sclerosis Model Cell Lines

**DOI:** 10.3390/cells10123554

**Published:** 2021-12-16

**Authors:** Sana Latif, Young-Sook Kang

**Affiliations:** College of Pharmacy and Drug Information Research Institute, Sookmyung Women’s University, 100 Cheongpa-ro 47-gil, Yongsan-gu, Seoul 04310, Korea; its.sanairfan@gmail.com

**Keywords:** L-arginine, cationic amino acid transporter, nitric oxide synthase, amyotrophic lateral sclerosis, NSC-34 cell lines

## Abstract

L-Arginine, a semi-essential amino acid, was shown to delay dysfunction of motor neurons and to prolong the lifespan, upon analysis of transgenic mouse models of amyotrophic lateral sclerosis (ALS). We investigated the transport function of arginine and neuronal nitric oxide synthase (nNOS) expression after pretreatment with L-arginine in NSC-34 hSOD1^WT^ (wild-type, WT) and hSOD1^G93A^ (mutant-type, MT) cell lines. [^3^H]L-Arginine uptake was concentration-dependent, voltage-sensitive, and sodium-independent in both cell lines. Among the cationic amino acid transporters family, including system y+, b^0,+^, B^0,+^, and y^+^L, system y^+^ is mainly involved in [^3^H]L-arginine transport in ALS cell lines. System b^0,+^ accounted for 23% of the transport in both cell lines. System B^0,+^ was found only in MT, and whereas, system y^+^L was found only in WT. Lysine competitively inhibited [^3^H]L-arginine uptake in both cell lines. The nNOS mRNA expression was significantly lower in MT than in WT. Pretreatment with arginine elevated nNOS mRNA levels in MT. Oxidizing stressor, H_2_O_2_, significantly decreased their uptake; however, pretreatment with arginine restored the transport activity in both cell lines. In conclusion, arginine transport is associated with system y^+^, and neuroprotection by L-arginine may provide an edge as a possible therapeutic target in the treatment of ALS.

## 1. Introduction

Amyotrophic lateral sclerosis (ALS) is a neurodegenerative disease with most cases being sporadic; however, 5–10% are familial [1]. ALS is caused by mutations in more than 50 disease-modifying genes, most prevalent ones include mutations in copper/zinc superoxide dismutase type-1 (SOD1), chromosome 9 open reading frame 72 (C9orf72), TAR DNA-binding, and fused in sarcoma (FUS), which account for 25% of familial ALS cases [2].

Recently, researchers have shown increased interest in amino acid profiling and its potential application in the early diagnosis of diverse chronic diseases, such as Alzheimer’s disease (AD), dementia, ALS, Parkinson’s disease (PD), and Huntington’s disease (HD) [3]. Previous studies have suggested that the generalized defect in the excitatory amino acids metabolism in the ALS results in their altered distribution between intracellular and extracellular pools, resulting in the interference of excitatory transmission carried by glutamate receptors that can lead to degeneration of motor neurons [4]. The levels of free amino acids in the lumbar spinal cord of ALS mice were altered when compared with those in healthy mice [5]. In ALS disease model cell lines, D- and L-serine uptake rates differed between mutant (MT, NSC34/hSOD1^G93A^) and wild-type (WT, NSC34/hSOD1wt) cell lines [6]. Similarly, alterations in the transport rates of other essential amino acids, such as L-tryptophan, have been studied in NSC-34 cell lines, and results indicated that the uptake rate of [^3^H]L-tryptophan was lower in the MT than in the control cell lines [7].

L-Arginine is a conditionally essential amino acid derived from diet, protein breakdown, and de novo arginine production [8]. Human and preclinical studies have illustrated the effects of nutrition as disease-modifying in ALS [9]. L-Arginine plays a role targeting the pathophysiological mechanism of ALS. L-Arginine enhances the blood flow leading to protein synthesis and ɑ- ketoglutarate generation [9]. Arginine and ɑ- ketoglutarate might have beneficial effects in ALS by improving growth of skeletal muscle, reducing the defects of glucose metabolism in skeletal muscle, and sustaining the production of energy for muscle function [10]. In addition, arginine has shown in vitro protection of the skeletal muscle cells from wasting in NO independent and mechanistic target of rapamycin complex 1 (mTORC1) dependent manner [11]. Arginine deficiency makes neurons sensitive to excitotoxic injury. Previous study has shown that arginine plays a protective role in motor neuron against the neurotoxicity induced by glutamate in motor neuron primary culture [1].

The cellular availability of arginine is determined by the capacity and efficiency of transporters expressed in the plasma membrane and is mostly mediated by members of the solute carrier 7 family (SLC7), which is divided into two subgroups: cationic amino acid transporters (CATs, SLC7A1-4) and L-type amino acid transporters (LATs) [12]. Substrates of cationic amino acids (CAA) such as L-arginine, L-lysine, L-histidine and L-ornithine are transported distinctively by four transport systems such as system y+ (Slc7a1), b^0,+^ (Slc7a9), B^0,+^ (Slc6a14) and y^+^L (Slc7a7) [13,14]. The identification and characterization of these transport systems rely on Na^+^ dependency, kinetics, and cationic amino acid specificity [15]. System y^+^ mainly carried the transport of CAA in a sodium independent manner and is also voltage sensitive [16]. The electrical component in the system y^+^ is the driving force for transmembrane potential for the influx of CAA [17]. System B^0,+^, is the only sodium dependent transporter involve in the transport of CAA and neutral amino acids (NAA). System b^0,+^, found in mouse blastocytes, is sodium independent transporter that carries CAA and NAA [18]. Study in humans have shown that deficiency in the system b^0,+^ leads to cystinuria [19] and system y^+^L in erythrocytes leads to the lysinuric intolerance [20]. 

Nitric oxide (NO), a versatile molecule generated in the signaling and non-specific immune defense processes, is involved in the transport and catabolism of arginine, ultimately contributing to the delicate balance between the normal and pathological outcomes of NO production [21]. The major forms of NOS encoded by three separate genes include neuronal (nNOS/NOS1), inducible (iNOS/NOS2), and endothelial NOS (eNOS/NOS3). Although nitrogen tyrosylation of macromolecules and NO causes motor neuron death, it is unclear which type of NOS is specifically involved in the pathogenesis of ALS [22]. Previous research has shown that NO levels are increased in mtSOD1 (G93A) ALS mice (MT mice), with iNOS gene expression being markedly induced in astrocytes; however, nNOS gene expression is significantly reduced in the motor neurons of MT mice [23]. Superoxide is a free radical generated in many biological processes that can act as a precursor to other reactive oxygen species, including superoxide anions, hydroxyl radicals, and hydrogen peroxide (H_2_O_2_) [24], and can oxidatively damage lipids, proteins, and DNA, causing various diseases [25]. Earlier research has shown that L-arginine supplementation significantly boosted the host defenses by exerting its immunostimulatory effects in patient with breast cancer and tumor-bearing mice [26]. In another study, administration of L-arginine to mtSOD1 (G93A) ALS transgenic mice markedly delayed the lumbar spinal cord neuropathology and motor dysfunction onset hence prolonging the life span [1].

Our study aimed to evaluate the transport characteristics of arginine in ALS cell lines and to investigate nNOS expression. Using quantitative real-time polymerase chain reaction (qPCR) analysis, we further sought to investigate nNOS regulation after treating NSC-34 cell lines with arginine and citrulline. In addition, we also investigated the effect of [^3^H]L-arginine uptake under neuroinflammatory conditions.

## 2. Materials and Methods

### 2.1. Radiolabelled Compounds and Chemicals

A total of 45.1 Ci/mmol of [^3^H]L-arginine was purchased from PerkinElmer (Boston, MA, USA). Other reagent-grade chemicals and drugs were purchased from Sigma Aldrich (St. Louis, MO, USA).

### 2.2. Cell Culture

NSC-34 is a motor neuron-like hybrid cell line produced from mouse neuroblastoma fused with an embryonic mouse primary spinal cord cells [27]. The NSC-34 cell lines were provided by Prof. Hoon Ryu (KIST, Seoul, Korea) and were categorized into wild-type hSOD1 (NSC-34/hSOD1^WT^ cells, WT) and mutant-type hSOD1^G93A^ (NSC-34/hSOD1^G93A^ cells, MT). Cells were cultured (WT passage number 8–24 and MT passage number 5–19) in accordance with previously described procedures [5]; seeded onto rat tail collagen type-I-coated culture plates; and grown in a high-glucose-containing medium (Hyclone, Salt Lake City, UT, USA) supplemented with 10% fetal bovine serum (Hyclone), containing 100 U/mL penicillin and 100 µg/mL streptomycin. The NSC-34 cell lines were subsequently incubated at 37 °C in a humidified atmosphere with 5% CO_2_/95% air.

### 2.3. [^3^H]L-Arginine Uptake Study in ALS Model Cell Lines

The [^3^H]L-arginine uptake study in NSC-34 cells was conducted according to previously described methods [28]. Briefly, NSC-34 cell lines in 24-well plates were washed thrice with extracellular fluid (ECF; pH 7.4). After adding 200 μL ECF buffer containing [^3^H]L-arginine (55.4 nM, 0.5 µCi/well) to each well, cell lines were incubated for 5 min at 37 °C. Next, ice-cold ECF buffer was used to terminate [^3^H]L-arginine uptake. Thereafter, 750 µL of 1 N NaOH was used to solubilize the cells overnight at room temperature. Radioactivity was measured using a liquid scintillation counter (LS6500; Beckman, Fullerton, CA, USA). The cell-to-medium ratio (µL/mg protein) was calculated as follows: [^3^H] radioactivity (dpm/µL) in the sample per milligram of cellular protein (dpm/mg protein) [29].

Similarly, in the sodium and ion replacement uptake study, ECF buffer was prepared by replacing sodium chloride with lithium chloride and choline chloride to disrupt the Na^+^ gradient and K^+^ chloride to disrupt the membrane potential ion gradient. The contribution of CAT transport systems to arginine uptake was measured using different buffer solutions: (a) media containing sodium; (b) sodium (lithium)-free media; (c) sodium-free media containing 5 mM alanine; (d) media containing sodium and 5 mM leucine [30].

### 2.4. Kinetic Analysis of [^3^H]L-Arginine in ALS Model Cell Lines

For saturation kinetics, the initial uptake of [^3^H]L-arginine at 0–1 mM was calculated using the Michaelis–Menten constant (K_m_) and maximum uptake rate (V_max_) as shown in the following equation 1:

V = [V_max1_ × C/(K_m1_ + C) + K_ns1_ × C] + [V_max2_ × C/(K_m2_ + C) + K_ns2_ × C], (1)

where C is arginine concentration; V_max1_ and V_max2_ are the maximum uptake rates for high- and low-affinity processes, respectively; K_m1_ and K_m2_ are the Michaelis–Menten constants for the high- and low-affinity processes, respectively; and K_ns1_ and K_ns2_ are first-order constants for the non-saturable components in the high- and low-affinity processes, respectively.

### 2.5. qPCR Analysis of CAT-1(Slc7a1) and nNOS (Nos1) mRNA Expression in NSC-34 Cells

Using the RNeasy Mini Kit (Qiagen, Valencia, CA, USA), total RNA was isolated from the cultured NSC-34 cell lines according to the manufacturer’s guidelines [31]. Next, 2 µg total RNA was reverse-transcribed using a high-capacity cDNA reverse transcription kit (Applied Biosystems, Foster City, CA, USA) in a gene amplification system (Mycycler; Bio-Rad Laboratories, Hercules, CA, USA). qPCR was performed according to the manufacturer’s protocol in a 48-well plate using the StepOnePlus PCR system (Applied Biosystems). Subsequently, the TaqMan probe primer sequence for CAT-1 (Slc7a1) was obtained from Invitrogen (Carlsbad, CA, USA) with the sense and antisense sequences 5′-CTTGGGCGCTGGTGTCTATG-3′ and 5′-CGTAGCTGTAGAGGTAGGCTG-3′, respectively. The primers for nNOS (Nos1) had the sense and antisense sequences 5′-CCCAACGTCATTTCTGTCCGT-3′ and 5′-TCTACCAGGGGCCGATCATT-3′, respectively. The housekeeping gene *GAPDH* was used as the loading control [32].

### 2.6. Statistical Data Analysis

All data are presented as the mean ± standard error of the mean (SEM). Data were analyzed using SigmaPlot (version 12; Systat Software Inc., Richmond, CA, USA). One-way ANOVA with Dunnett’s post hoc test and an unpaired two-tailed Student’s *t*-test were used to determine treatment differences. Statistical significance was set at *p* < 0.05.

## 3. Results

### 3.1. Kinetic Parameters of the [^3^H]L-Arginine Uptake Is Altered in ALS Cell Lines Model

**Figure 1:**
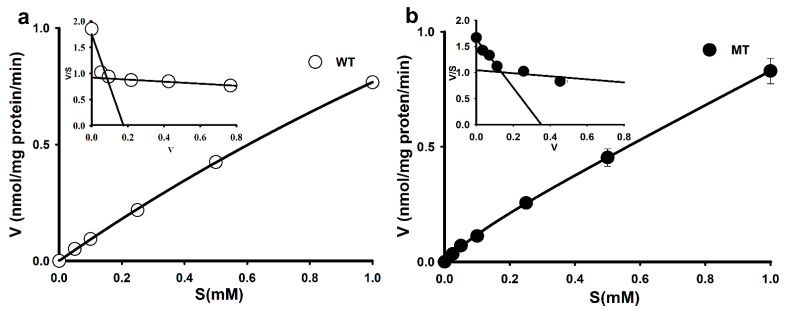
Concentration-dependent [^3^H]L-arginine uptake in ALS cell lines. (**a**) Saturation kinetics of [^3^H]L-arginine in the WT cell line. (**b**) Saturation kinetics of [^3^H]L-arginine in the MT cell line. Uptake was measured after incubation for 5 min at 37 °C and pH 7.4 in the presence or absence of 0–1 mM unlabeled L-arginine. Each point represents the mean ± SEM. (*n* = 3–4). The inset graphs are presented as Eadie–Hofstee Scatchard plots.

To determine the L-arginine transport mechanism in the ALS model, MT was used as the disease model and WT as a control model cell lines. [^3^H]L-Arginine uptake was found to be concentration-dependent in NSC-34 cell lines; however, its uptake in the MT cell lines was lower than that in the WT cell lines (Figure 1). 

Additional analysis using an Eadie–Hofstee plot yielded two distinct slopes (Figure 1a,b, inset graph), indicating that two saturable components were involved in arginine uptake in WT and MT cell lines. The Michaelis–Menten constant (K_m_) and maximum uptake rate (V_max_) were fitted to the Equation (1), and at the high-affinity site, the affinity (K_m_) was 23-fold lower and the capacity (V_max_) was approximately 40-fold higher in the MT cell lines than in the WT cell lines. However, at the low-affinity site, both affinity and capacity in the MT cell lines did not markedly differ from those in the WT cell lines (Table 1).

Overall, the results revealed that the MT cell lines had an altered affinity and capacity for arginine transport.

### 3.2. Differential Analysis of Arginine Transport Systems in ALS Cell Lines

[^3^H]L-Arginine uptake was found to be Na^+^-independent in both WT and MT cell lines, as shown in Table 2. 

Using the differential analysis technique, the contribution of each system was calculated. For instance, the contribution of system B^o,+^ was determined by subtracting the uptake of arginine in Na^+^-containing media from Na^+^-free media (Table 3). 

Our data revealed that among the various CAT transporter systems, in MT cell lines; system B^o,+^ contributes to 9.8%; however, system B^o,+^ had no contribution in the WT cell lines. The contribution of system b^o,+^ in the transport of [^3^H]L-arginine in both NSC-34 cell lines was approximately 23%. Although system y^+^L was not identified in MT cell lines, a 3.4% contribution to [^3^H]L-arginine uptake was observed in WT cell lines. In conclusion, our data showed that system y^+^ contributed most significantly to [^3^H]L-arginine transport, with 74 and 68% contributions in WT and MT cell lines, respectively (Table 3).

### 3.3. Modulation of the [^3^H]L-Arginine Uptake by Transporter Inhibitors in ALS Cell Lines

The arginine transport mechanism was further investigated using several compounds and transporter inhibitors. [^3^H]L-Arginine uptake was significantly inhibited in NSC-34 cell lines by arginine, lysine, histidine, ornithine, leucine, and alanine. Glutamine and choline were found to have no inhibitory effect on [^3^H]L-arginine uptake (Table 4). 

In addition, the L-arginine analog N-methyl-L-arginine (L-NMMA) strongly inhibited [^3^H]L-arginine uptake by 32 and 50% in the WT and MT cell lines, respectively, indicating that uptake was mediated by system y^+^. Homoarginine, which is associated with system y^+^, significantly inhibited [^3^H]L-arginine uptake in NSC-34 cell lines. Similarly, harmaline, a system b^o,+^ inhibitor, also showed significant [^3^H]L-arginine uptake inhibition. Furthermore, N-ethylmaleimide (NEM), a system y^+^ specific inhibitor, significantly inhibited the uptake in both cell lines, showing sensitivity to system y^+^. N-methylmaleimide (NMM), which is system y^+^L inhibitor, also showed significant inhibition in both cell lines. However, 2-aminobicyclo-(2,2,1)-heptane-2-carboxylic acid (BCH), a system B^o,+^ inhibitor, showed no inhibition in either cell line (Table 4).

### 3.4. Inhibitory Effect of the [^3^H]L-Arginine Uptake by Various Drugs in ALS Model Cell Lines

The inhibitory effect of various drugs on [^3^H]L-arginine transport was scrutinized in NSC-34 cell lines. Quinidine, an antiarrhythmic drug, significantly inhibited [^3^H]L-arginine uptake at a concentration of 2 mM. Notably, [^3^H]L-arginine uptake was inhibited by more than 50% in the presence of gabapentin in both cell lines, whereas verapamil showed 68 and 73% inhibition in MT and WT cell lines, respectively. Clonidine and donepezil did not inhibit [^3^H]L-arginine uptake in either cell lines (Table 5).

**Figure 2:**
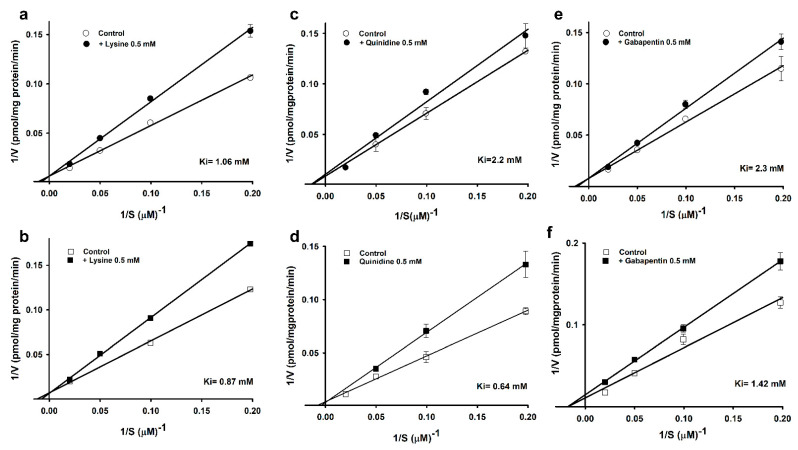
Line weaver–Burk plot of [^3^H]L-arginine uptake by ALS cell lines. [^3^H]L-arginine uptake was observed at pH 7.4 and 37 °C for 5 min in the presence of 0.5 mM of lysine (**a**,**b**), quinidine (**c**,**d**), and gabapentin (**e**,**f**) or their absence in WT (circle) and MT (square) cell lines. Each data point represents the mean ± SEM (*n* = 3–4).

Furthermore, in our study, the Lineweaver–Burk plot demonstrated high-affinity uptake at varying L-arginine concentrations (5–50 µM) in the presence or absence of 500 µM lysine, quinidine, and gabapentin. In the case of lysine, the two lines of the plot intersected at the ordinate, indicating that lysine competitively inhibited L-arginine (Figure 2a,b).

Quinidine showed non-competitive inhibition in the WT cell lines, with a Ki value of 2.2 mM, and competitive inhibition in the MT cell lines, with a Ki value of 0.64 mM (Figure 2c,d). In addition, gabapentin showed competitive inhibition in the WT and non-competitive inhibition in the MT cell lines, with Ki values of 2.3 and 1.42 mM (Figure 2e,f), respectively. Our data indicated that the MT cell lines were more sensitive than the control (Figure 2).

### 3.5. Effect of Arginine Pretreatment on nNOS mRNA Expression Levels in ALS Cell Lines

**Figure 3:**
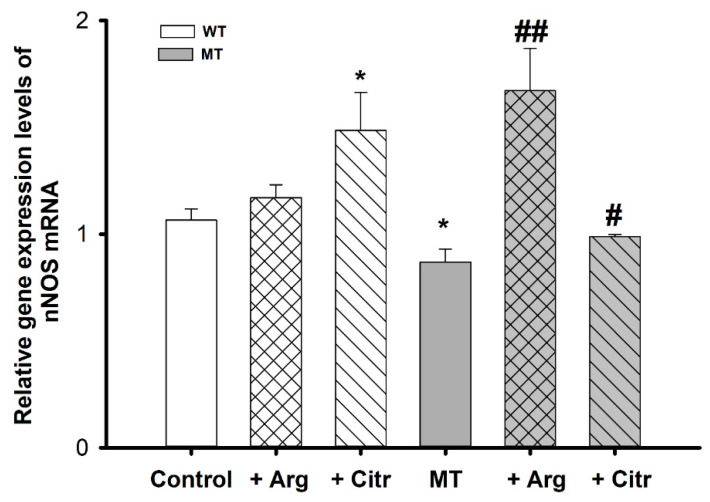
nNOS mRNA expression levels in ALS cell lines measured using qPCR. Each value represents the mean ± SEM (*n* = 3–4). * *p* < 0.05, indicate significant differences with respect to the control. # *p* < 0.05, ## *p* < 0.01, significant differences vs. MT.

To assess the pattern of nNOS levels, we measured mRNA expression using qPCR in WT and MT cell lines, with or without the addition of arginine and citrulline. nNOS mRNA expression was lower in the MT cell lines than in the control; however, nNOS relative gene expression significantly increased in MT cell lines treated with arginine and citrulline, in comparison to untreated MT cell lines. In the WT cell lines, there was no statistical difference in the mRNA expression between the untreated and arginine-treated cells. In contrast, citrulline treatment resulted in a significant increase in nNOS expression compared with that in untreated WT cell lines (Figure 3).

### 3.6. Neuroprotective Effects of Arginine against H_2_O_2_ Motor Neuronal Cytotoxicity

**Figure 4:**
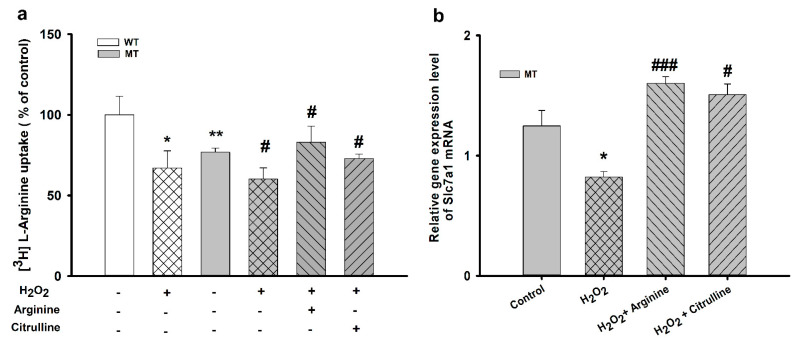
Pretreatment effect of oxidative stress-inducing H_2_O_2_ on [^3^H]L-arginine uptake by ALS cell lines**.** ALS cell lines were exposed to 100 µM H_2_O_2_ for 24 h. (**a**) [^3^H]L-Arginine uptake was measured at pH 7.4 and 37 °C for 5 min in WT and MT cell lines pretreated with H_2_O_2_ with or without treatment with 10 mM L-arginine and L-citrulline. (**b**) mRNA expression level of Slc7a1 in the MT cell line. Each value represents the mean ± SEM (*n* = 3–4). * *p* < 0.05, ** *p* < 0.01, significant differences vs. control; # *p* < 0.05, ### *p* < 0.001, vs. H_2_O_2_ treatment.

Changes in [^3^H]L-arginine uptake upon preincubation of NSC-34 cell lines with 100 µM H_2_O_2_ are shown in Figure 4. 

H_2_O_2_ significantly decreased [^3^H]L-arginine uptake in both cell lines compared with that in control. Additionally, 10 mM of arginine and citrulline increased [^3^H]L-arginine transport rate in the MT cell lines. The Slc7a1 mRNA expression was markedly decreased in the presence of H_2_O_2_ alone in the MT cell lines; however, in addition to co-treatment with H_2_O_2_ and arginine, co-treatment with H_2_O_2_ and citrulline also resulted in a significant increase in the [^3^H]L-arginine uptake rate. These results suggest the restorative and neuroprotective roles of arginine and citrulline in ALS cell lines.

## 4. Discussion

L-Arginine, one of the CAT-1 substrates, is a precursor of nitric oxide (NO) and polyamines and is involved in various biochemical pathways, such as protein synthesis and the urea cycle [33].For the treatment of ALS, no effective therapy or cure is available. Although the exact mechanism of ALS disease is not fully known, neuroinflammation and microglia activation plays a role in the progression of disease [34]. Previously L-arginine has been shown to protect cultured motor neurons against excitotoxic injury [1]. In a current study, we aimed to evaluate the transport characteristics of L-arginine in ALS cell lines and show the effect of arginine on nNOS expression. NSC-34 cell lines are mouse motor neuron-like cells containing human SOD1 (NSC-34/hSOD1^wt^) and SOD1 (G93A) gene mutation and are widely used for the study of ALS disease mechanisms and also to discover the therapeutically effective drug regimen in motor neurodegeneration [32,35]. Arginine uptake in NSC-34 cells occurred in a concentration-dependent manner, indicating a carrier-mediated transport system (Figure 1). The Eadie–Hofstee plot indicated that two saturable processes are involved in arginine transport in NSC-34 cell lines. Furthermore, the parameters indicated in Table 1 show that at the high-affinity site, affinity (K_m_) was lower and capacity (V_max_) higher in the MT cell lines than those in the WT cell lines. Conversely, at low-affinity sites, higher affinity and lower capacity were observed in the MT cell lines than in the WT cell lines. These results are consistent with arginine uptake in TR-iBRB2 cell lines [13], suggesting that L-arginine transport occurs via a carrier-mediated transport system rather than passive diffusion in ALS cell lines. Taking these findings into consideration might explain the relatively lower expression of transporters in disease model. The lower uptake of L-arginine in the MT cell line compared to WT cell line might be due to deleted or mutated transporter systems. A similar phenomenon was shown in the previous research work in phenyl butyric acid in NSC-34 cell lines [32]. [^3^H]L-Arginine uptake occurs in a Na^+^-independent manner, ruling out the idea of a Na^+^-dependent transport system such as system B^0,+^ in NSC-34 cell lines. In addition, [^3^H]L-arginine uptake was voltage-sensitive (Table 2), consistent with the findings of Bae et al., who reported decreased arginine uptake in KCl-containing media during experimental uptake in CAD cells [30].

The paucity of information on arginine uptake in NSC-34 cell lines prompted us to further examine the transporter types involved in L-arginine transport in NSC-34 cell lines. Arginine is known to be transported by major four cationic amino acid transporters including system y^+^, b^0,+^, B^0,+^, and y^+^L. Previous studies have also shown that system y^+^ is an arginine-specific transporter associated with several cell types, including neurons [30,36,37,38]. In our differential analysis (Table 3), we found that system y^+^ is primarily involved in arginine transport, and Slc7a1 is expressed in NSC-34 cell lines, as shown in our previous work [39]. Furthermore, our results suggest that system b^0,+^ is involved, to a lesser extent, in L-arginine transport in MT and WT cell lines, although mRNA expression has not been observed in NSC-34 cell lines [39]. Earlier studies have showed L-arginine transport by both systems y^+^L and y^+^ in human umbilical vein endothelial cells [40]; and in the uptake of arginine in the CAD cells [30], however, our data showed no involvement of system y^+^L in the MT cell lines, whereas only 3.4% involvement was observed in the WT cell lines. Therefore, we hypothesize that the transport of arginine in the ALS model cell line is carried mainly by system y^+^. In our study, it has been shown that the [^3^H]L-arginine uptake was significantly inhibited by typical CAT-1 substrates, including arginine, lysine, histidine, and ornithine, in both cell lines (Table 4). These results were comparable to those for TR-iBRB2 cell lines [13], and the blood-retinal and blood-brain barrier [41]. This might be due to the fact that these CAA follows the same transport systems. In addition, [^3^H]L-arginine uptake was remarkably reduced in both cell lines by L-NMMA, a potent and competitive non-specific NOS inhibitor that competes with arginine in cellular transport and for the NOS active site [8]. The co-expression of two transport systems, such as systems y^+^ and y^+^L, can be identified through their differential sensitivity to the sulfhydryl reagents of N-ethylmaleimide (NEM) [42]. NEM, is a typical inhibitor of system y^+^ and our data revealed significant inhibition of [^3^H]L-arginine transport due to NEM in both cell lines, leading to the hypothesis that system y^+^ was primarily involved in arginine transport in these cell lines (Table 4). However, partial inhibition also suggests the contribution of other transporter systems, strengthening our data in Table 3. Earlier research in K562 cells showed a similar pattern of our results [33].

In our study, quinidine significantly inhibited [^3^H]L-arginine uptake in ALS model cell lines. Earlier studies have shown the effectiveness of quinidine combined with dextromethorphan in treating pseudobulbar affect, a consequence of neurological damage caused by ALS, AD, and other traumatic brain injuries, overall the combination has shown to improve the quality of life of ALS patients [43]. In addition, verapamil, a calcium channel blocker, has shown to delay the onset of ALS disease, increase the lifespan in SOD1(G93A) mice [44]. In our study, verapamil significantly inhibited [^3^H]L-arginine uptake in ALS model cell lines (Table 5). Furthermore, in the Lineweaver–Burk plot analysis, the high-affinity uptake of [^3^H]L-arginine was competitively inhibited by lysine, which is also a substrate of CAT-1, suggesting that the high-affinity uptake of arginine and lysine both shared the same transport process which is system y^+^. Therefore, showed competitive inhibition in both cell lines. Similar results have been reported previously on arginine, in which it was competitively inhibited by ornithine, another CAT-1 substrate [13]. Interestingly, quinidine, showed noncompetitive inhibition in WT and competitive inhibition in MT cell line. Ki value in MT cell line showed that diseased model cell line is sensitive to inhibition. The non- competitive inhibition in WT cell line might be because quinidine which is a substrate of p-glycoprotein follows the transporter other than the CAT-1 transporter. Surprisingly, gabapentin showed competitive inhibition in WT and non-competitive inhibition in MT cell lines (Figure 2). The competitive inhibition in WT showed sensitivity towards system y^+^ and the non-competitive inhibition in MT cell line suggest that both arginine and gabapentin follows different transport systems and therefore, we hypothesize that both drugs can be co- administered in the disease state. Earlier findings have shown that gabapentin follows a large neutral amino acids, L-type transporter [45], and has an inhibition pattern, indicating that transporters comprising systems B^o,+^, b^o,+^, and y^+^ have a strong overlapping specificity with system L [16].

L-Arginine can be metabolized into various biological metabolites, including NO, polyamines, proline, and agmatine. Of these, NO plays a pivotal role in several physiological and pathological conditions, including glutamate-induced neuronal death in ALS [46]. Our data revealed significantly lower nNOS mRNA expression in the MT than in the WT cell lines. Arginine and citrulline treatment resulted in increased nNOS mRNA and gene expression compared with those in untreated MT cell lines (Figure 3). Our results are consistent with those of a previous study that showed lower nNOS mRNA expression in the lumbar spinal cord of ALS mice compared with that in WT mice [22]. Previous studies have shown that L-citrulline is produced as coproduct in the synthesis of NO from arginine and plays role as a precursor for the biosynthesis of L-arginine in vascular cells [47], and citrulline plays role in the numerous physiological regulations such as inflammation and gene expression [28,48]. Furthermore, it has been shown that the supplementation of arginine to SOD1 (G93A) ALS mice has shown to reduce neuronal loss and preservation of nNOS levels near to normal control levels [22]. In addition, reduced nNOS levels have been demonstrated in ALS muscle in a study by Sorarù et al. [49].

In ALS, protein aggregation, inflammatory pathways, and oxidative stress are considered the proposed pathological mechanisms [23]. As reported by earlier that for neurotoxic study, NSC-34 cells plays a key characteristic role as they respond to the low doses of neurotoxins such as glutamate, TNF-α, and H_2_O_2_ [27]_._ [^3^H]L-arginine uptake was markedly reduced in the presence of H_2_O_2_ in NSC-34 cell lines, which might be attributed to the reduced expression of the transporters involved in arginine transport in ALS cell lines. However, arginine and citrulline pretreatment significantly increased arginine uptake in the MT cell lines, indicating that arginine and citrulline restored arginine uptake by restoring the expression of transporters. Our qPCR analysis revealed a similar pattern of results (Figure 4). Previous study on lysine uptake in NSC-34 cell lines has shown the similar results. The mRNA expression of SLC7a1 was depleted with oxidizing stress inducing agent H_2_O_2_ and was recovered by lysine. [39]. Overall, these results suggest a neuroprotective effect of arginine in the ALS model cell lines, consistent with other studies showing the effective role of arginine [1], and outlines the relationship between the downregulation of CAT-1 arginine transporters under inflammatory conditions [8].

## 5. Conclusions

In conclusion, our study revealed that arginine transport was mainly mediated by system y^+^ in NSC-34 cell lines and that two saturable transport processes were involved in both NSC-34 cell lines. Additionally, arginine exhibited regulatory effects, restoring nNOS expression in the disease model cell lines, and a neuroprotective effect against the oxidizing stress-inducing agent H_2_O_2_. Therefore, by understanding the transport system involved in arginine transport using motor neuron models, such as NSC-34 cell lines, L-arginine may act as a potential candidate for improving the pathological conditions in ALS and other neurodegenerative diseases.

## Figures and Tables

**Table 1:** Pharmacokinetic parameters of [^3^H]L-arginine uptake in ALS cell lines.

Parameters	WT	MT
K_m1_ (mM)	0.013 ± 0.005	0.30 ± 0.11 ***
K_m2_ (mM)	3.51 ± 1.73	1.98 ± 1.10
V_max1_(nmol/mg protein/min)	0.012 ± 0.006	0.47 ± 0.15 **
V_max2_(nmol/mg protein/min)	3.30 ± 1.62	1.42 ± 1.30

K_m_ and V_max_ are the transport affinity and maximum transport velocity, respectively. ** *p* < 0.01 and *** *p* < 0.001 indicate significant differences with respect to the control (WT). Each value represents the mean ± S.E.M. (*n* = 3).

**Table 2:** Sodium-dependency and membrane potential effects on the uptake of [^3^H]L-arginine by ALS cell lines.

Treatment	Relative Uptake (% of Control)	
	WT	MT
Control	100 ± 5	100 ± 1
**Na^+^ replacement**		
Lithium chloride	97.1 ± 0.4	90.0 ± 5.3
Chlorine Chloride	122 ± 10	97.9 ± 6.0
**Membrane potential**		
Potassium chloride	71.2 ± 7.9 *	89.9 ± 1.9 *

[^3^H]L-Arginine uptake by ALS cell lines was performed at 37 °C and pH 7.4 for 5 min. Each value represents the mean ± S.E.M. (*n* = 3–4). * *p* < 0.05 indicates a significant difference with respect to the control.

**Table 3:** Relative contribution of transporters involved in the uptake of [^3^H]L-arginine in ALS cell lines.

Treatment	Transport Systems	L-Arginine Uptake (Cell/Medium Ratio) (µL/mg Protein)		Detection of Transport System	Relative Contribution (Cell/Medium Ratio) (µL/mg Protein)	
		**WT**	MT		WT	MT
(a) Na^+^-containing	y^+^; y^+^L, B^o,+^ b^o,+^	13.9 ± 0.31	8.33 ± 0.01	(a) − (b) = B^o,+^	-	1.0 ± 0.1 (9.8%)
(b) Na^+^ free	y^+^; y^+^L, b^o,+^	14.6 ± 0.4	7.4 ± 0.8	(b) − (c) = b^o,+^	3.3 ± 0.4 (23%)	2.3 ± 0.71 (23%)
(c) Na^+^ free plus alanine	y^+^; y^+^L	11.3 ± 0.1	5.1 ± 0.4	(c) − (d) = y^+^L	0.49 ± 0.06 (3.4%)	-
(d) Na^+^-containing plus leucine	y^+^	10.8 ± 1.3	6.9 ± 0.3	(d) = y^+^	11 ± 1 (74%)	6.9 ± 0.3 (68%)

[^3^H]L-Arginine uptake by WT and MT cell lines was measured at 37 °C and pH 7.4 for 5 min in the presence (control) or absence of sodium (Na^+^). In (c) and (d), the Na^+^-free buffer contains 5 mM of alanine and leucine. Each value represents the mean ± S.E.M. (*n* = 3–4).

**Table 4:** Effect of several amino acids and transporter inhibitors on the uptake of [^3^H]L-arginine by ALS cell lines.

Substrate	Conc.(mM)	Uptake of [^3^H]L-Arginine (% of Control)	
		WT	MT
Control		100 ± 5	100 ± 7
+Arginine	2	33.9 ± 3.8 ***	37.2 ± 5.9 ***
+Lysine	2	65.9 ± 3.7 **	52.9 ± 3.8 ***
+Histidine	2	75.5 ± 0.2 **	69.7 ± 6.8 **
+Ornithine	2	72.0 ± 6.3 **	79.0 ± 1.5 **
+Leucine	2	68.2 ± 1.7 **	43.9 ± 4.7 ***
+Alanine	2	72.7 ± 4.5 *	78.9 ± 6.2 *
+Glutamine	2	104 ± 8	113 ± 4
+Choline	2	119 ± 7	116 ± 4
+NMMA	2	32.1 ± 1.9 ***	50.0 ± 2.0 **
+Homoarginine	2	41.6 ± 1.4 ***	65.7 ± 6.3 ***
+Harmaline	2	76.3 ± 10.0 *	65.2 ± 3.4 *
+NMM	2	63.6 ± 9.3 **	69.7 ± 7.2 **
+NEM	2	79.3 ± 5.7 **	68.5 ± 1.0 ***
+BCH	2	93.5 ± 5.3	85.2 ± 5.5

[^3^H]L-Arginine uptake in the absence (control) or presence of 2 mM inhibitor solutions was performed at 37 °C and pH 7.4 for 5 min. Each value represents the mean ± S.E.M. (*n* = 3–4). * *p* < 0.05, ** *p* < 0.01, and *** *p* < 0.001 indicate significant differences with respect to the control. NMMA, N-monomethyl-L-arginine; NMM, N-methylmaleimide; NEM, N-ethylmaleimide; BCH, 2-aminobicyclo-(2,2,1)-heptane-2-carboxylic acid.

**Table 5:** Inhibitory effect of various pharmacological drugs on [^3^H]L-arginine uptake by ALS cell lines.

Substrates	Conc. (mM)	[^3^H]L-Arginine Uptake (% of Control)	
		WT	MT
Control		100 ± 6	100 ± 5
+Quinidine	2	76.8 ± 5.1 **	52.8 ± 6.1 ***
+Verapamil	0.5	73.6 ± 9.5 *	67.1 ± 3.0 **
+Gabapentin	2	56.8 ± 1.2 ***	58.9 ± 7.4 **
+Clonidine	2	81.2 ± 5.9	109 ± 9
+Donepezil	2	97.5 ± 2.9	109 ± 9

[^3^H] L-Arginine uptake was performed in the absence (control) or presence of the indicated drug solutions (0.5–2 mM) at 37 °C and pH 7.4 for 5 min. Each value represents the mean ± S.E.M. (*n* = 3–4). * *p* < 0.05, ** *p* < 0.01, and *** *p* < 0.001 indicate significant differences with respect to the control.

## Data Availability

Not applicable.
